# Out‐of‐pocket fees for health care in Australia: implications for equity

**DOI:** 10.5694/mja2.51895

**Published:** 2023-04-02

**Authors:** Emily J Callander

**Affiliations:** ^1^ University of Technology Sydney Sydney NSW

**Keywords:** Economics, medical, Health financing, Health policy, Health systems, Healthcare disparities, Socioeconomic status

Out‐of‐pocket fees create access barriers to health care, exacerbating health inequalities

In Australia, 15% of all expenditure on health care comes directly from individuals in the form of out‐of‐pocket fees — this is almost double the amount contributed by private health insurers.^1^ There is concern that vulnerable groups — socio‐economically disadvantaged people and older Australians in particular, who also have higher health care needs — are spending larger proportions of their incomes on out‐of‐pocket fees for health care.^2^ A 2019 study identified that one in three low income households are spending more than 10% of their income on health care.^3^ This might create economic hardship, and individuals do forgo care,^4^ with one in four Australians without a health care condition and up to one in two with certain health conditions avoiding care because of the cost.^4^

Health care services in Australia are delivered through a mixture of public and private providers, with governments subsidising the costs of care but out‐of‐pocket fees remaining a significant component.^5^ Australia is not unique in this, with similar systems in New Zealand, Ireland, France, Germany, the Netherlands, and the United Kingdom. However, in Australia, out‐of‐pocket fees make up a larger proportion of overall health expenditure than in these other countries.^6^ The amount paid by households on health care in Australia was estimated to be $3200 in 2014,^3^ with out‐of‐pocket fees per health care service rising over time.^7^ The increasing out‐of‐pocket expenditure by patients is concerning in light of international experience in the United States, where there is a reliance on private or market‐based health care, and health care costs are the leading cause of bankruptcy.^8^

The level of out‐of‐pocket fees in Australia has ignited vigorous policy and academic debate.^9^, ^10^, ^11^ Varied viewpoints range from the impact of high fees on a patient's ability to access care^4^ and the equity implications of high fees,^12^ to the right of private providers to set their own fees in an open market and to recover costs of providing care.^13^ Out‐of‐pocket fees are also part of cost‐sharing measures between governments and patients, as a result of increasing government expenditure on health care and unprecedented levels of demand.^1^ This article examines the current provision of health care and out‐of‐pocket fees within Australia through a micro‐economic lens, identifying the access and equity implications of the dual public–private system, and considers potential systems‐level options for a way forward.

## Universal health care and private health insurance in Australia

Under Australia's universal health care system, individuals can access care in public hospitals free of charge. Public hospitals are owned and operated by state governments. Outside of public hospitals, health care services are owned and operated by private providers on either a for‐profit or not‐for‐profit basis. The costs to individuals for accessing these services are partly subsidised by the federal government through Medicare. Medicare covers services such as consultations with general practitioners and specialists, and diagnostic tests and imaging.

Australia also has numerous policy incentives and penalties to encourage Australians to take out private health insurance and access private hospitals, and thus private specialist health care. Private health insurance covers the hospital stay component in private hospitals. For the actual health services provided by private specialists within private hospitals, Medicare will pay a subsidy for the service, with an individual's private health insurance potentially paying for either the remainder of the charge, or patients themselves also having to pay. This will depend upon the coverage of each individual policy, and the amount charged by the provider of the service, with many private health insurance policies only providing reimbursement up to a certain amount. Only 44% of private hospital admissions had no out‐of‐pocket fees in the 2020–21 financial year;^14^ and in the same period only 34% of specialist attendances were bulk billed (meaning there was no out‐of‐pocket fee).^7^ The average out‐of‐pocket fee for out‐of‐hospital specialist and obstetric services was $98 and $303 per non‐bulk billed visit, respectively.^7^ Out‐of‐pocket fees are therefore a major feature of private specialist care.

## User fees and the role of the market in setting price

Private health care services (ie, all services outside of public hospital services) are provided through the market. This means that the fee charged for services covers the cost of production (staff salaries, capital costs, and operation costs such as insurance) less any government subsidies. For providers operating on a for‐profit basis, it also includes a profit component, and the objective of such providers is profit maximisation. A recent report found that profits for private specialists increased by 11% between 2019–20 and 2020–21; profits for GPs increased by a smaller amount (2%).^13^ It is also notable that the average salary, before tax and after deducting practice costs, is around $400 000 per year for specialists, and around $200 000 per year for GPs.^15^

The ability of private providers of health care services to set their own fees, to cover operational costs and make profits, is a key feature of the Australian health care system. This is supported by the Australian Constitution, with government excluded from regulating fees that health care providers charge for their services.^16^ The fee charged, and the amount of profit, is therefore determined by an individual consumer's willingness to pay for the service. In the market, the higher the willingness to pay, the higher the service fee. This is problematic in health care as willingness to pay is constrained by ability to pay, with people at socio‐economic disadvantage — who generally have poorer health^17^ — having a lower ability to pay the higher prices often paid by those at socio‐economic advantage.^18^

Although the private market is subsidised through Medicare, patients are only reimbursed a fixed amount based on the Medicare schedule fee for each service. This schedule fee generally differs from the fees actually charged.^7^ The Medicare safety net reimburses patients at a higher amount (initially 85% for most out‐of‐hospital services, or 100% of GP services; increasing to 100% under the safety net) once they have reached a certain threshold of out‐of‐pocket expenditure in a year ($531.70 in March 2023). However, the disconnect between the schedule fee and the fees charged by providers still leaves patients vulnerable to open‐ended out‐of‐pocket fees (Box). The extended Medicare safety net applies when a higher threshold (in March 2023, $770 for people who have a concession card or family tax benefit, and $2414 for others) reimburses patients at 80% of out‐of‐pocket expenditure based on the actual provider fee; however, again this still leaves patients to pay a potentially high out‐of‐pocket amount.

Box 1 Vulnerability of patients to high out‐of‐pocket fees even with the Medicare safety net*

**Figure:**
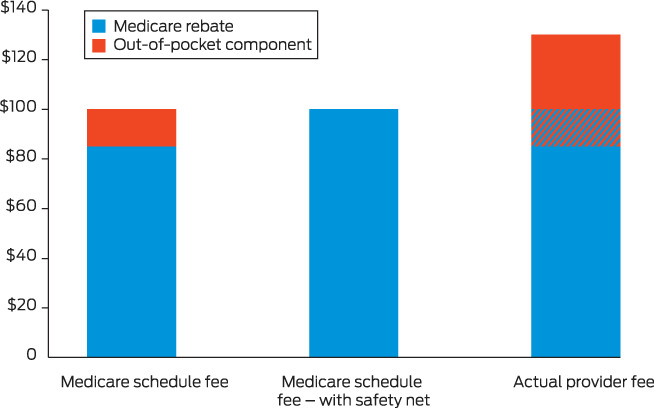
* Medicare schedule fee hypothetically set at $100 and actual provider fee hypothetically set at $130. These different amounts lead to patients being vulnerable to high out‐of‐pocket fees even with the Medicare safety net: $45 initially, or $30 with the Medicare safety net.

## Implications for access to care

With the market as the mechanism for the distribution of private care, only those with the ability to pay the market price will be able to access this care. To some extent, as a result of the dual private and public system in Australia, those who are unable to afford to pay or who are unwilling to pay the market price for private care may still be able to receive care through the public hospital system — with public hospitals providing care for all essential acute medical services, based upon urgency. However, this does not cover primary care, and waiting times in public hospitals for non‐urgent reasons might mean that people priced out of the private market are not able to achieve access. Using Queensland public hospital outpatient specialist clinics as an example, 20% of non‐urgent cardiac patients and 30% of non‐urgent respiratory patients wait more the 365 days to receive care.^19^

## Market undermining equity

Although people with higher incomes may have the ability to pay to access private specialist care, such user fees cannot themselves directly contribute to the promotion of equity. There is no direct transfer of out‐of‐pocket fees from people of higher socio‐economic status to those of lower socio‐economic status. Out‐of‐pocket fees, and by extension government subsidies, do nothing to directly subsidise access for people of lower socio‐economic status who are unable to pay market prices. This, combined with public subsidisation of private health insurance premiums ($6.2 billion per year)^1^ means that there is potentially a large transfer of public expenditure (Medicare subsidies for private specialist care, plus private health insurance subsidies) to wealthier people and away from lower socio‐economic status groups,^20^ who are more likely to be in need of care.^17^ Allowing the more affluent to exercise their higher ability to pay only contributes to higher inequality by allowing higher socio‐economic status groups to access care more frequently.^21^, ^22^, ^23^

## Systems‐level options for change

A potential option to reduce out‐of‐pocket fees and reduce affordability barriers is for the federal government to expand Medicare coverage to areas such as dental, and increase the subsidies paid through Medicare, by increasing Medicare Benefit Schedule fees. However, previous increases in Medicare rebates have not resulted in substantial out‐of‐pocket cost reductions.^24^

Increasing the volume of outpatient specialist care through public hospitals might be an additional option to improving equity. Although there is a skew towards higher socio‐economic status in access to Medicare services,^21^, ^22^, ^23^, ^25^ public hospitals achieve greater equity in the provision of care than private hospitals.^25^, ^26^ However, public hospitals play a vital role contributing to equity in health access once conditions arise; they do not cover primary and preventive care.

The Pharmaceutical Benefits Scheme has also achieved equity in health care access.^27^ The Pharmaceutical Benefits Scheme differs from Medicare in that the federal government pays a set, agreed price to providers (pharmaceutical companies), and there is a maximum out‐of‐pocket price that consumers will pay for any medication. Introducing a low ceiling out‐of‐pocket fee under Medicare, whereby individuals never pay more than this amount for health care services and governments pay an agreed amount to providers, could produce more equitable access.

Many other options for change have also been proposed, such as incentives for bulk‐billed private specialist services, promoting greater price transparency, and funding specific conditions in bundles of funding (rather than based on frequency of services).^28^, ^29^, ^30^ There is therefore a considerable suite of options for reform.

## Conclusion

Out‐of‐pocket fees in Australia are already leading to patients avoiding care because of the cost. The US offers a salient reminder of the impacts of unaffordable health care. There are numerous options for reducing out‐of‐pocket fees and promoting affordability. Moving forward with active, bold reform should be a priority to ensure promotion of equity and truly universal health care in Australia.

## Open access

Open access publishing facilitated by University of Technology Sydney, as part of the Wiley ‐ University of Technology Sydney agreement via the Council of Australian University Librarians.

## Competing interests

No relevant disclosures.

## Provenance

Commissioned; externally peer reviewed.
